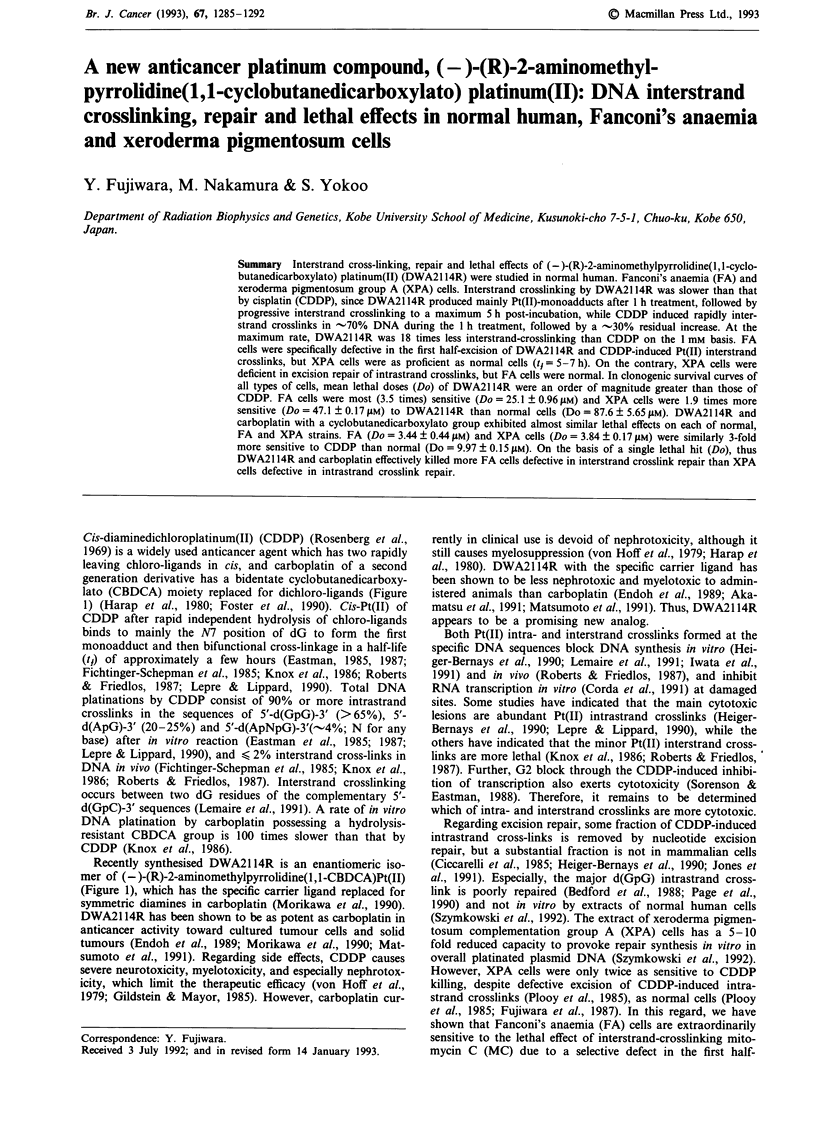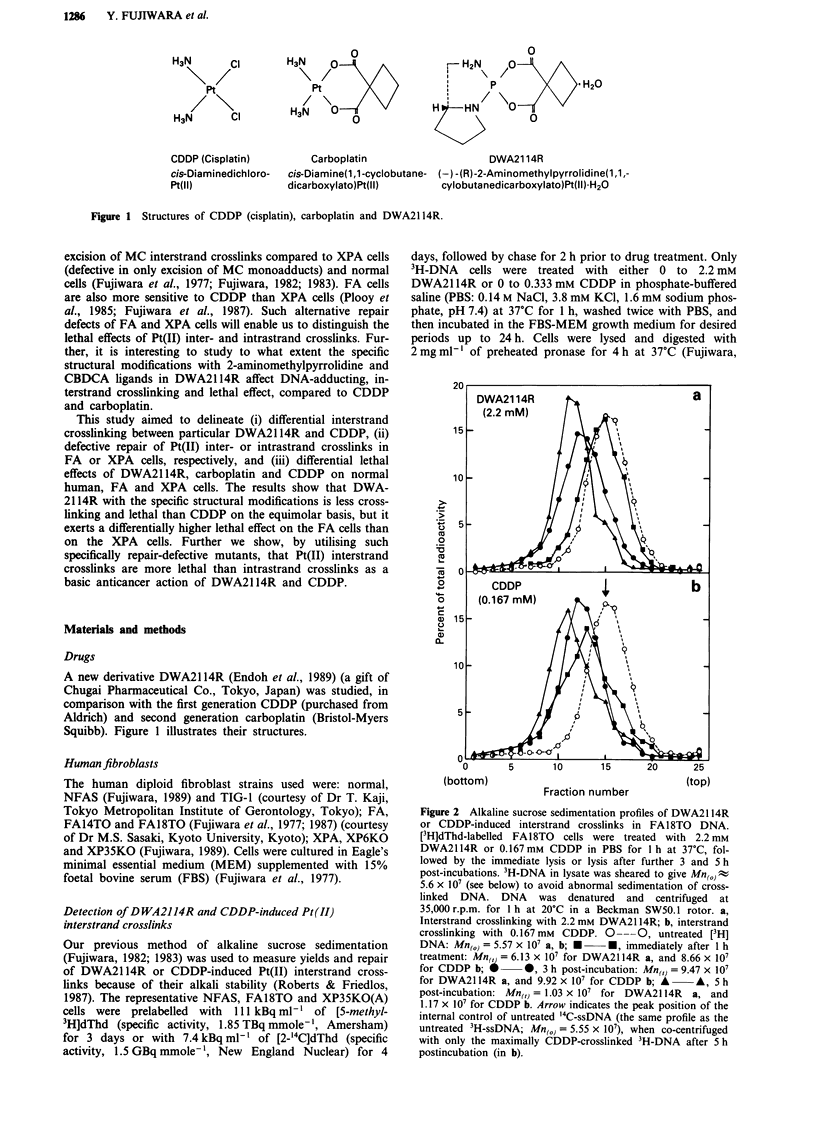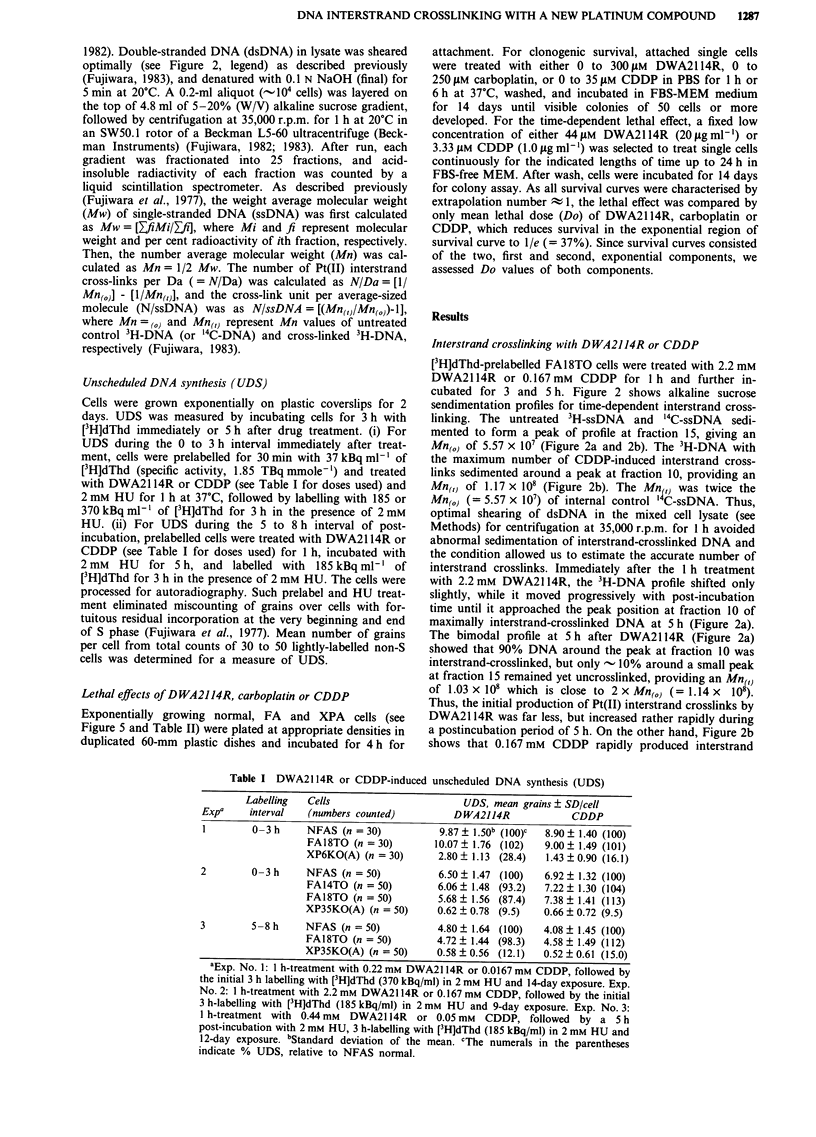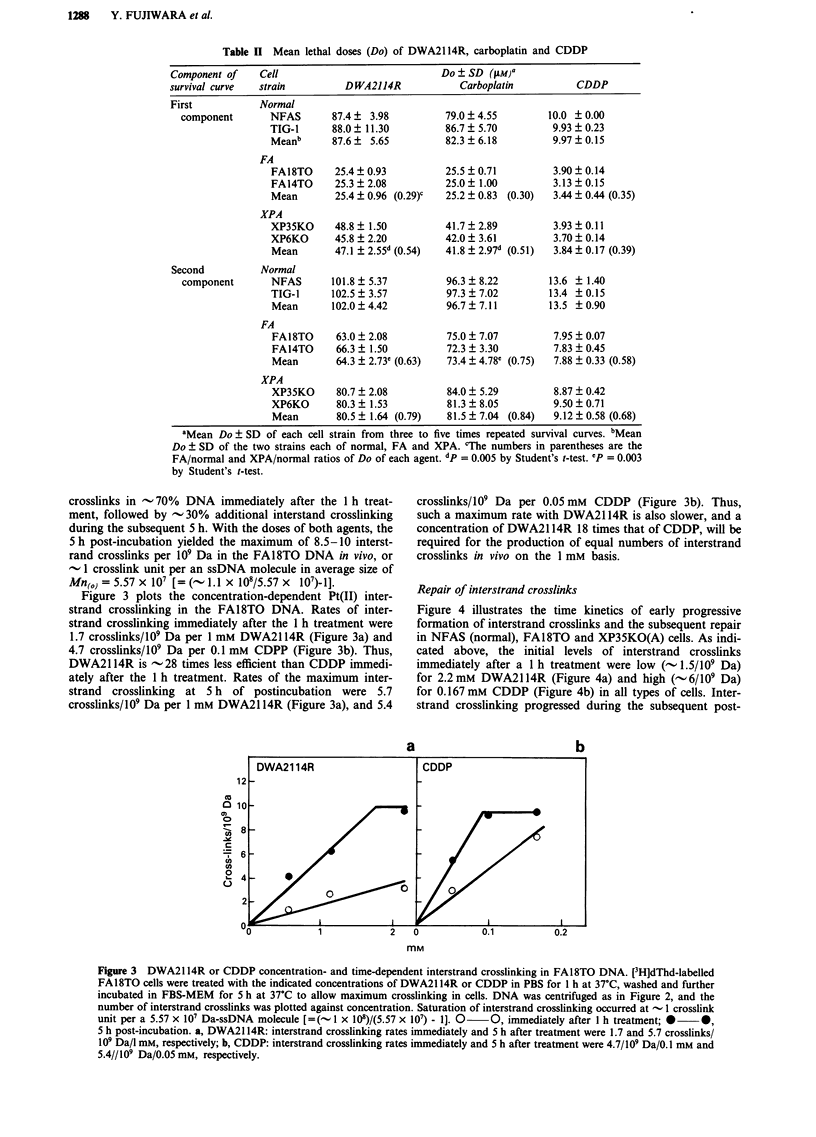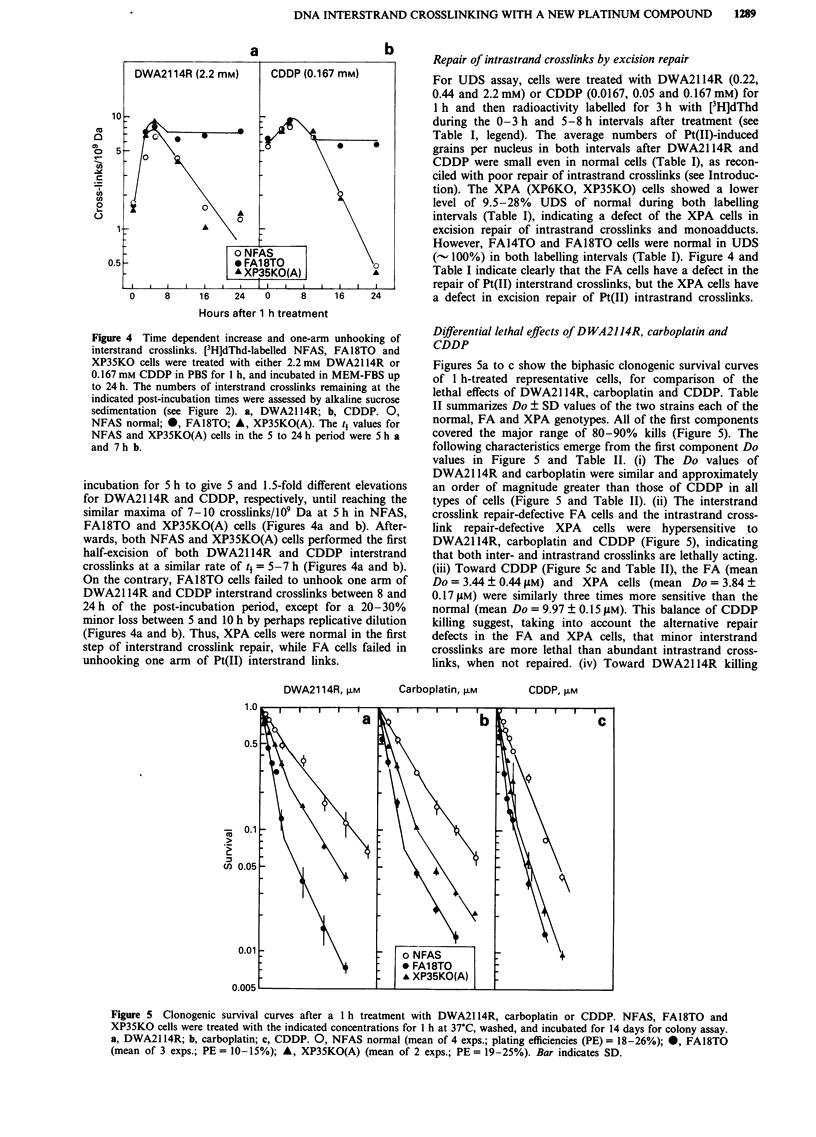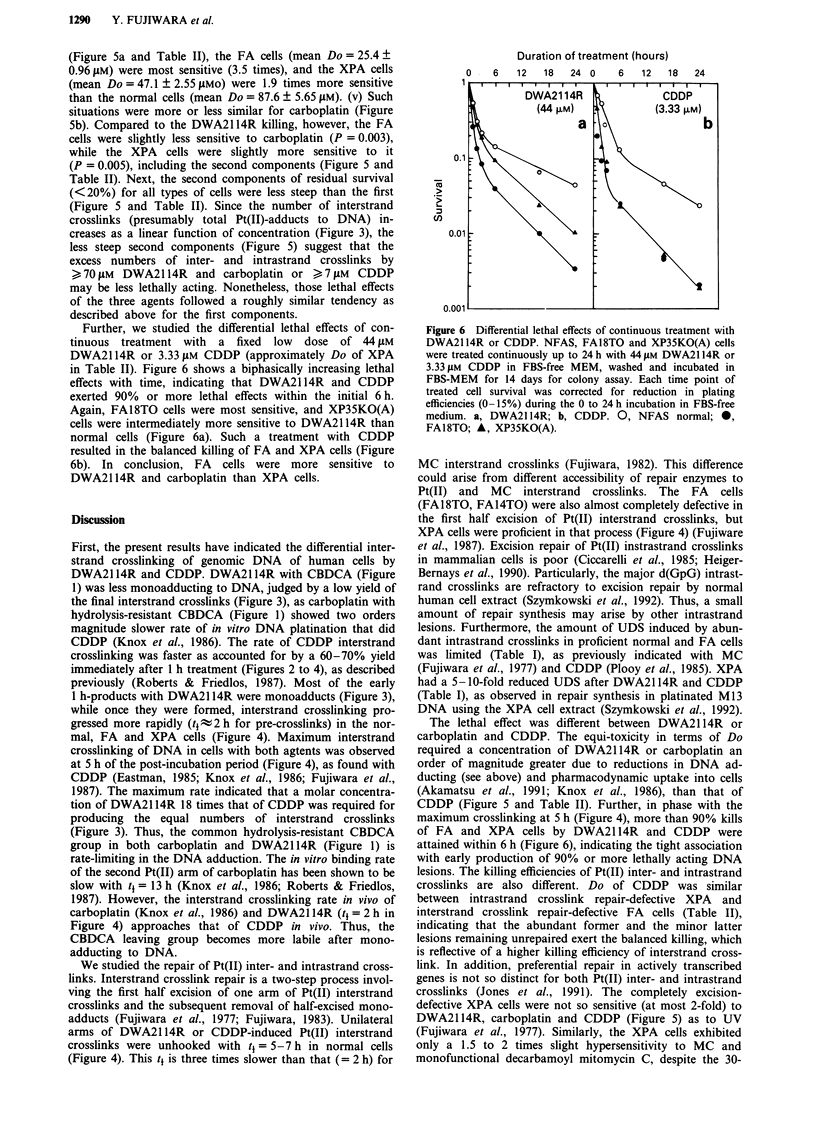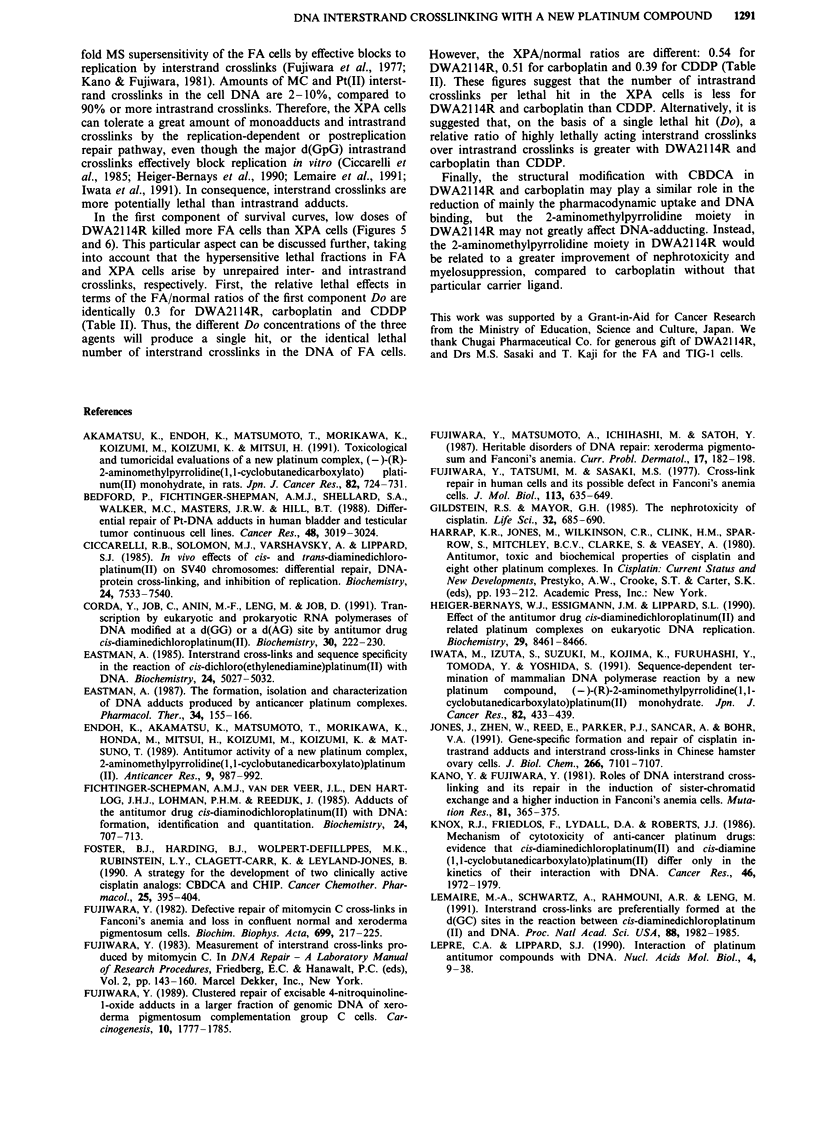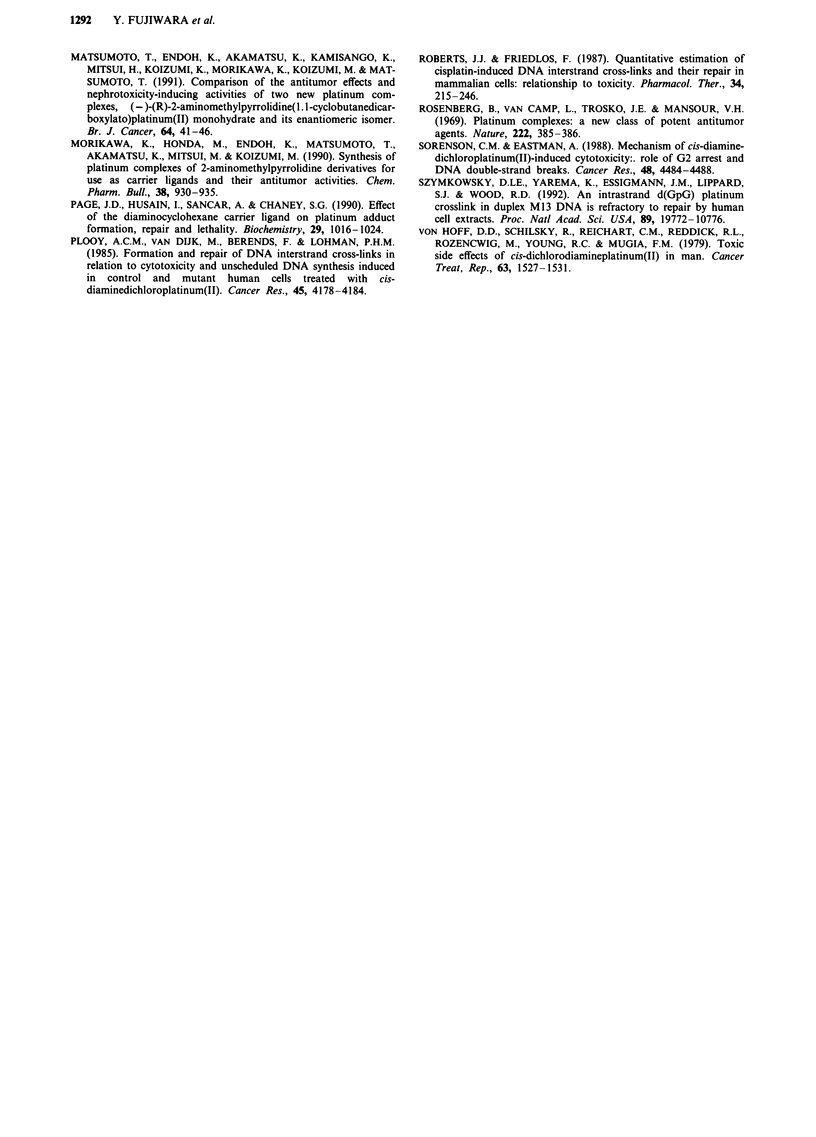# A new anticancer platinum compound, (-)-(R)-2-aminomethyl-pyrrolidine(1,1-cyclobutanedicarboxylato) platinum(II): DNA interstrand crosslinking, repair and lethal effects in normal human, Fanconi's anaemia and xeroderma pigmentosum cells.

**DOI:** 10.1038/bjc.1993.239

**Published:** 1993-06

**Authors:** Y. Fujiwara, M. Nakamura, S. Yokoo

**Affiliations:** Department of Radiation Biophysics and Genetics, Kobe University School of Medicine, Japan.

## Abstract

Interstrand cross-linking, repair and lethal effects of (-)-(R)-2-aminomethylpyrrolidine(1,1-cyclobutanedicarboxylato++ +) platinum(II) (DWA2114R) were studied in normal human. Fanconi's anaemia (FA) and xeroderma pigmentosum group A (XPA) cells. Interstrand crosslinking by DWA2114R was slower than that by cisplatin (CDDP), since DWA2114R produced mainly Pt(II)-monoadducts after 1 h treatment, followed by progressive interstrand crosslinking to a maximum 5 h post-incubation, while CDDP induced rapidly interstrand crosslinks in approximately 70% DNA during the 1 h treatment, followed by a approximately 30% residual increase. At the maximum rate, DWA2114R was 18 times less interstrand-crosslinking than CDDP on the 1 mM basis. FA cells were specifically defective in the first half-excision of DWA2114R and CDDP-induced Pt(II) interstrand crosslinks, but XPA cells were as proficient as normal cells (t1/2 = 5-7 h). On the contrary, XPA cells were deficient in excision repair of intrastrand crosslinks, but FA cells were normal. In clonogenic survival curves of all types of cells, mean lethal doses (Do) of DWA2114R were an order of magnitude greater than those of CDDP. FA cells were most (3.5 times) sensitive (Do = 25.1 +/- 0.96 microM) and XPA cells were 1.9 times more sensitive (Do = 47.1 +/- 0.17 microM) to DWA2114R than normal cells (Do = 87.6 +/- 5.65 microM). DWA2114R and carboplatin with a cyclobutanedicarboxylato group exhibited almost similar lethal effects on each of normal, FA and XPA strains. FA (Do = 3.44 +/- 0.44 microM) and XPA cells (Do = 3.84 +/- 0.17 microM) were similarly 3-fold more sensitive to CDDP than normal (Do = 9.97 +/- 0.15 microM). On the basis of a single lethal hit (Do), thus DWA2114R and carboplatin effectively killed more FA cells defective in interstrand crosslink repair than XPA cells defective in intrastrand crosslink repair.


Br. J. Cancer (1993), 67, 1285–1292 © Macmillan Press Ltd., 1993

### A new anticancer platinum compound, (−)-(R)-2-aminomethyl-pyrrolidine(1,1-cyclobutanedicarboxylato) platinum(II): DNA interstrand crosslinking, repair and lethal effects in normal human, Fanconi's anaemia and xeroderma pigmentosum cells

Y. Fujiwara, M. Nakamura & S. Yokoo

*Department of Radiation Biophysics and Genetics, Kobe University School of Medicine, Kusunoki-cho 7-5-1, Chuo-ku, Kobe 650, Japan.*

**Summary** Interstrand cross-linking, repair and lethal effects of (−)-(R)-2-aminomethylpyrrolidine(1,1-cyclobutanedicarboxylato) platinum(II) (DWA2114R) were studied in normal human. Fanconi's anaemia (FA) and xeroderma pigmentosum group A (XPA) cells. Interstrand crosslinking by DWA2114R was slower than that by cisplatin (CDDP), since DWA2114R produced mainly Pt(II)-monoadducts after 1 h treatment, followed by progressive interstrand crosslinking to a maximum 5 h post-incubation, while CDDP induced rapidly interstrand crosslinks in ~70% DNA during the 1 h treatment, followed by a ~30% residual increase. At the maximum rate, DWA2114R was 18 times less interstrand-crosslinking than CDDP on the 1 mM basis. FA cells were specifically defective in the first half-excision of DWA2114R and CDDP-induced Pt(II) interstrand crosslinks, but XPA cells were as proficient as normal cells ($t_{\frac{1}{2}}$ = 5–7 h). On the contrary, XPA cells were deficient in excision repair of intrastrand crosslinks, but FA cells were normal. In clonogenic survival curves of all types of cells, mean lethal doses (*Do*) of DWA2114R were an order of magnitude greater than those of CDDP. FA cells were most (3.5 times) sensitive (*Do* = 25.1 ± 0.96 μM) and XPA cells were 1.9 times more sensitive (*Do* = 47.1 ± 0.17 μM) to DWA2114R than normal cells (Do = 87.6 ± 5.65 μM). DWA2114R and carboplatin with a cyclobutanedicarboxylato group exhibited almost similar lethal effects on each of normal, FA and XPA strains. FA (*Do* = 3.44 ± 0.44 μM) and XPA cells (*Do* = 3.84 ± 0.17 μM) were similarly 3-fold more sensitive to CDDP than normal (Do = 9.97 ± 0.15 μM). On the basis of a single lethal hit (*Do*), thus DWA2114R and carboplatin effectively killed more FA cells defective in interstrand crosslink repair than XPA cells defective in intrastrand crosslink repair.

*Cis*-diaminedichloroplatinum(II) (CDDP) (Rosenberg *et al.*, 1969) is a widely used anticancer agent which has two rapidly leaving chloro-ligands in *cis*, and carboplatin of a second generation derivative has a bidentate cyclobutanedicarboxylato (CBDCA) moiety replaced for dichloro-ligands (Figure 1) (Harap *et al.*, 1980; Foster *et al.*, 1990). *Cis*-Pt(II) of CDDP after rapid independent hydrolysis of chloro-ligands binds to mainly the *N7* position of dG to form the first monoadduct and then bifunctional cross-linkage in a half-life ($t_{\frac{1}{2}}$) of approximately a few hours (Eastman, 1985, 1987; Fichtinger-Schepman *et al.*, 1985; Knox *et al.*, 1986; Roberts & Friedlos, 1987; Lepre & Lippard, 1990). Total DNA platinations by CDDP consist of 90% or more intrastrand crosslinks in the sequences of 5′-d(GpG)-3′ (>65%), 5′-d(ApG)-3′ (20–25%) and 5′-d(ApNpG)-3′(~4%; N for any base) after *in vitro* reaction (Eastman *et al.*, 1985; 1987; Lepre & Lippard, 1990), and ⩽ 2% interstrand cross-links in DNA *in vivo* (Fichtinger-Schepman *et al.*, 1985; Knox *et al.*, 1986; Roberts & Friedlos, 1987). Interstrand crosslinking occurs between two dG residues of the complementary 5′-d(GpC)-3′ sequences (Lemaire *et al.*, 1991). A rate of *in vitro* DNA platination by carboplatin possessing a hydrolysis-resistant CBDCA group is 100 times slower than that by CDDP (Knox *et al.*, 1986).

Recently synthesised DWA2114R is an enantiomeric isomer of (−)-(R)-2-aminomethylpyrrolidine(1,1-CBDCA)Pt(II) (Figure 1), which has the specific carrier ligand replaced for symmetric diamines in carboplatin (Morikawa *et al.*, 1990). DWA2114R has been shown to be as potent as carboplatin in anticancer activity toward cultured tumour cells and solid tumours (Endoh *et al.*, 1989; Morikawa *et al.*, 1990; Matsumoto *et al.*, 1991). Regarding side effects, CDDP causes severe neurotoxicity, myelotoxicity, and especially nephrotoxicity, which limit the therapeutic efficacy (von Hoff *et al.*, 1979; Gildstein & Mayor, 1985). However, carboplatin currently in clinical use is devoid of nephrotoxicity, although it still causes myelosuppression (von Hoff *et al.*, 1979; Harap *et al.*, 1980). DWA2114R with the specific carrier ligand has been shown to be less nephrotoxic and myelotoxic to administered animals than carboplatin (Endoh *et al.*, 1989; Akamatsu *et al.*, 1991; Matsumoto *et al.*, 1991). Thus, DWA2114R appears to be a promising new analog.

Both Pt(II) intra- and interstrand crosslinks formed at the specific DNA sequences block DNA synthesis *in vitro* (Heiger-Bernays *et al.*, 1990; Lemaire *et al.*, 1991; Iwata *et al.*, 1991) and *in vivo* (Roberts & Friedlos, 1987), and inhibit RNA transcription *in vitro* (Corda *et al.*, 1991) at damaged sites. Some studies have indicated that the main cytotoxic lesions are abundant Pt(II) intrastrand crosslinks (Heiger-Bernays *et al.*, 1990; Lepre & Lippard, 1990), while the others have indicated that the minor Pt(II) interstrand cross-links are more lethal (Knox *et al.*, 1986; Roberts & Friedlos, 1987). Further, G2 block through the CDDP-induced inhibition of transcription also exerts cytotoxicity (Sorenson & Eastman, 1988). Therefore, it remains to be determined which of intra- and interstrand crosslinks are more cytotoxic.

Regarding excision repair, some fraction of CDDP-induced intrastrand cross-links is removed by nucleotide excision repair, but a substantial fraction is not in mammalian cells (Ciccarelli *et al.*, 1985; Heiger-Bernays *et al.*, 1990; Jones *et al.*, 1991). Especially, the major d(GpG) intrastrand cross-link is poorly repaired (Bedford *et al.*, 1988; Page *et al.*, 1990) and not *in vitro* by extracts of normal human cells (Szymkowski *et al.*, 1992). The extract of xeroderma pigmentosum complementation group A (XPA) cells has a 5–10 fold reduced capacity to provoke repair synthesis *in vitro* in overall platinated plasmid DNA (Szymkowski *et al.*, 1992). However, XPA cells were only twice as sensitive to CDDP killing, despite defective excision of CDDP-induced intrastrand crosslinks (Plooy *et al.*, 1985), as normal cells (Plooy *et al.*, 1985; Fujiwara *et al.*, 1987). In this regard, we have shown that Fanconi's anaemia (FA) cells are extraordinarily sensitive to the lethal effect of interstrand-crosslinking mitomycin C (MC) due to a selective defect in the first half-

Correspondence: Y. Fujiwara.
Received 3 July 1992; and in revised form 14 January 1993.

CDDP (Cisplatin) *cis*-Diaminedichloro-Pt(II)

Carboplatin *cis*-Diamine(1,1-cyclobutane-dicarboxylato)Pt(II)

DWA2114R (–)-(R)-2-Aminomethylpyrrolidine(1,1,-cylobutanedicarboxylato)Pt(II)·$H_2O$

**Figure 1** Structures of CDDP (cisplatin), carboplatin and DWA2114R.

excision of MC interstrand crosslinks compared to XPA cells (defective in only excision of MC monoadducts) and normal cells (Fujiwara *et al.*, 1977; Fujiwara, 1982; 1983). FA cells are also more sensitive to CDDP than XPA cells (Plooy *et al.*, 1985; Fujiwara *et al.*, 1987). Such alternative repair defects of FA and XPA cells will enable us to distinguish the lethal effects of Pt(II) inter- and intrastrand crosslinks. Further, it is interesting to study to what extent the specific structural modifications with 2-aminomethylpyrrolidine and CBDCA ligands in DWA2114R affect DNA-adducting, interstrand crosslinking and lethal effect, compared to CDDP and carboplatin.

This study aimed to delineate (i) differential interstrand crosslinking between particular DWA2114R and CDDP, (ii) defective repair of Pt(II) inter- or intrastrand crosslinks in FA or XPA cells, respectively, and (iii) differential lethal effects of DWA2114R, carboplatin and CDDP on normal human, FA and XPA cells. The results show that DWA-2114R with the specific structural modifications is less crosslinking and lethal than CDDP on the equimolar basis, but it exerts a differentially higher lethal effect on the FA cells than on the XPA cells. Further we show, by utilising such specifically repair-defective mutants, that Pt(II) interstrand crosslinks are more lethal than intrastrand crosslinks as a basic anticancer action of DWA2114R and CDDP.

### Materials and methods

### *Drugs*

A new derivative DWA2114R (Endoh *et al.*, 1989) (a gift of Chugai Pharmaceutical Co., Tokyo, Japan) was studied, in comparison with the first generation CDDP (purchased from Aldrich) and second generation carboplatin (Bristol-Myers Squibb). Figure 1 illustrates their structures.

### *Human fibroblasts*

The human diploid fibroblast strains used were: normal, NFAS (Fujiwara, 1989) and TIG-1 (courtesy of Dr T. Kaji, Tokyo Metropolitan Institute of Gerontology, Tokyo); FA, FA14TO and FA18TO (Fujiwara *et al.*, 1977; 1987) (courtesy of Dr M.S. Sasaki, Kyoto University, Kyoto); XPA, XP6KO and XP35KO (Fujiwara, 1989). Cells were cultured in Eagle's minimal essential medium (MEM) supplemented with 15% foetal bovine serum (FBS) (Fujiwara *et al.*, 1977).

### *Detection of DWA2114R and CDDP-induced Pt(II) interstrand crosslinks*

Our previous method of alkaline sucrose sedimentation (Fujiwara, 1982; 1983) was used to measure yields and repair of DWA2114R or CDDP-induced Pt(II) interstrand crosslinks because of their alkali stability (Roberts & Friedlos, 1987). The representative NFAS, FA18TO and XP35KO(A) cells were prelabelled with 111 kBq $ml^{-1}$ of [5-*methyl*-$^3$H]dThd (specific activity, 1.85 TBq $mmole^{-1}$, Amersham) for 3 days or with 7.4 kBq $ml^{-1}$ of [2-$^{14}$C]dThd (specific activity, 1.5 GBq $mmole^{-1}$, New England Nuclear) for 4 days, followed by chase for 2 h prior to drug treatment. Only $^3$H-DNA cells were treated with either 0 to 2.2 mM DWA2114R or 0 to 0.333 mM CDDP in phosphate-buffered saline (PBS: 0.14 M NaCl, 3.8 mM KCl, 1.6 mM sodium phosphate, pH 7.4) at 37°C for 1 h, washed twice with PBS, and then incubated in the FBS-MEM growth medium for desired periods up to 24 h. Cells were lysed and digested with 2 mg $ml^{-1}$ of preheated pronase for 4 h at 37°C (Fujiwara,

**Figure 2** Alkaline sucrose sedimentation profiles of DWA2114R or CDDP-induced interstrand crosslinks in FA18TO DNA. [$^3$H]dThd-labelled FA18TO cells were treated with 2.2 mM DWA2114R or 0.167 mM CDDP in PBS for 1 h at 37°C, followed by the immediate lysis or lysis after further 3 and 5 h post-incubations. $^3$H-DNA in lysate was sheared to give $Mn_{(o)} \approx 5.6 \times 10^7$ (see below) to avoid abnormal sedimentation of crosslinked DNA. DNA was denatured and centrifuged at 35,000 r.p.m. for 1 h at 20°C in a Beckman SW50.1 rotor. **a**, Interstrand crosslinking with 2.2 mM DWA2114R; **b**, interstrand crosslinking with 0.167 mM CDDP. O---O, untreated [$^3$H] DNA: $Mn_{(o)} = 5.57 \times 10^7$ **a**, **b**; ■——■, immediately after 1 h treatment: $Mn_{(t)} = 6.13 \times 10^7$ for DWA2114R **a**, and $8.66 \times 10^7$ for CDDP **b**; ●——●, 3 h post-incubation: $Mn_{(t)} = 9.47 \times 10^7$ for DWA2114R **a**, and $9.92 \times 10^7$ for CDDP **b**; ▲——▲, 5 h post-incubation: $Mn_{(t)} = 1.03 \times 10^7$ for DWA2114R **a**, and $1.17 \times 10^7$ for CDDP **b**. *Arrow* indicates the peak position of the internal control of untreated $^{14}$C-ssDNA (the same profile as the untreated $^3$H-ssDNA; $Mn_{(o)} = 5.55 \times 10^7$), when co-centrifuged with only the maximally CDDP-crosslinked $^3$H-DNA after 5 h postincubation (in **b**).

1982). Double-stranded DNA (dsDNA) in lysate was sheared optimally (see Figure 2, legend) as described previously (Fujiwara, 1983), and denatured with 0.1 N NaOH (final) for 5 min at 20°C. A 0.2-ml aliquot ($\sim 10^4$ cells) was layered on the top of 4.8 ml of 5–20% (W/V) alkaline sucrose gradient, followed by centrifugation at 35,000 r.p.m. for 1 h at 20°C in an SW50.1 rotor of a Beckman L5-60 ultracentrifuge (Beckman Instruments) (Fujiwara, 1982; 1983). After run, each gradient was fractionated into 25 fractions, and acid-insoluble radiactivity of each fraction was counted by a liquid scintillation spectrometer. As described previously (Fujiwara *et al.*, 1977), the weight average molecular weight (*Mw*) of single-stranded DNA (ssDNA) was first calculated as $Mw = [\sum fiMi/\sum fi]$, where *Mi* and *fi* represent molecular weight and per cent radioactivity of *i*th fraction, respectively. Then, the number average molecular weight (*Mn*) was calculated as $Mn = 1/2\ Mw$. The number of Pt(II) interstrand cross-links per Da ( = N/Da) was calculated as $N/Da = [1/Mn_{(o)}] - [1/Mn_{(t)}]$, and the cross-link unit per average-sized molecule (N/ssDNA) was as $N/ssDNA = [(Mn_{(t)}/Mn_{(o)})-1]$, where $Mn =_{(o)}$ and $Mn_{(t)}$ represent *Mn* values of untreated control $^3$H-DNA (or $^{14}$C-DNA) and cross-linked $^3$H-DNA, respectively (Fujiwara, 1983).

### *Unscheduled DNA synthesis (UDS)*

Cells were grown exponentially on plastic coverslips for 2 days. UDS was measured by incubating cells for 3 h with [$^3$H]dThd immediately or 5 h after drug treatment. (i) For UDS during the 0 to 3 h interval immediately after treatment, cells were prelabelled for 30 min with 37 kBq ml$^{-1}$ of [$^3$H]dThd (specific activity, 1.85 TBq mmole$^{-1}$) and treated with DWA2114R or CDDP (see Table I for doses used) and 2 mM HU for 1 h at 37°C, followed by labelling with 185 or 370 kBq ml$^{-1}$ of [$^3$H]dThd for 3 h in the presence of 2 mM HU. (ii) For UDS during the 5 to 8 h interval of post-incubation, prelabelled cells were treated with DWA2114R or CDDP (see Table I for doses used) for 1 h, incubated with 2 mM HU for 5 h, and labelled with 185 kBq ml$^{-1}$ of [$^3$H]dThd for 3 h in the presence of 2 mM HU. The cells were processed for autoradiography. Such prelabel and HU treatment eliminated miscounting of grains over cells with fortuitous residual incorporation at the very beginning and end of S phase (Fujiwara *et al.*, 1977). Mean number of grains per cell from total counts of 30 to 50 lightly-labelled non-S cells was determined for a measure of UDS.

### *Lethal effects of DWA2114R, carboplatin or CDDP*

Exponentially growing normal, FA and XPA cells (see Figure 5 and Table II) were plated at appropriate densities in duplicated 60-mm plastic dishes and incubated for 4 h for attachment. For clonogenic survival, attached single cells were treated with either 0 to 300 μM DWA2114R, 0 to 250 μM carboplatin, or 0 to 35 μM CDDP in PBS for 1 h or 6 h at 37°C, washed, and incubated in FBS-MEM medium for 14 days until visible colonies of 50 cells or more developed. For the time-dependent lethal effect, a fixed low concentration of either 44 μM DWA2114R (20 μg ml$^{-1}$) or 3.33 μM CDDP (1.0 μg ml$^{-1}$) was selected to treat single cells continuously for the indicated lengths of time up to 24 h in FBS-free MEM. After wash, cells were incubated for 14 days for colony assay. As all survival curves were characterised by extrapolation number $\approx 1$, the lethal effect was compared by only mean lethal dose (*Do*) of DWA2114R, carboplatin or CDDP, which reduces survival in the exponential region of survival curve to $1/e$ ( = 37%). Since survival curves consisted of the two, first and second, exponential components, we assessed *Do* values of both components.

### Results

### *Interstrand crosslinking with DWA2114R or CDDP*

[$^3$H]dThd-prelabelled FA18TO cells were treated with 2.2 mM DWA2114R or 0.167 mM CDDP for 1 h and further incubated for 3 and 5 h. Figure 2 shows alkaline sucrose sedimentation profiles for time-dependent interstrand cross-linking. The untreated $^3$H-ssDNA and $^{14}$C-ssDNA sedimented to form a peak of profile at fraction 15, giving an $Mn_{(o)}$ of $5.57 \times 10^7$ (Figure 2a and 2b). The $^3$H-DNA with the maximum number of CDDP-induced interstrand cross-links sedimented around a peak at fraction 10, providing an $Mn_{(t)}$ of $1.17 \times 10^8$ (Figure 2b). The $Mn_{(t)}$ was twice the $Mn_{(o)}$ ( $= 5.57 \times 10^7$) of internal control $^{14}$C-ssDNA. Thus, optimal shearing of dsDNA in the mixed cell lysate (see Methods) for centrifugation at 35,000 r.p.m. for 1 h avoided abnormal sedimentation of interstrand-crosslinked DNA and the condition allowed us to estimate the accurate number of interstrand crosslinks. Immediately after the 1 h treatment with 2.2 mM DWA2114R, the $^3$H-DNA profile shifted only slightly, while it moved progressively with post-incubation time until it approached the peak position at fraction 10 of maximally interstrand-crosslinked DNA at 5 h (Figure 2a). The bimodal profile at 5 h after DWA2114R (Figure 2a) showed that 90% DNA around the peak at fraction 10 was interstrand-crosslinked, but only ~10% around a small peak at fraction 15 remained yet uncrosslinked, providing an $Mn_{(t)}$ of $1.03 \times 10^8$ which is close to $2 \times Mn_{(o)}$ ( $= 1.14 \times 10^8$). Thus, the initial production of Pt(II) interstrand crosslinks by DWA2114R was far less, but increased rather rapidly during a postincubation period of 5 h. On the other hand, Figure 2b shows that 0.167 mM CDDP rapidly produced interstrand

**Table I** DWA2114R or CDDP-induced unscheduled DNA synthesis (UDS)

| Exp[a] | Labelling interval | Cells (numbers counted) | UDS, mean grains ± SD/cell DWA2114R | CDDP |
|---|---|---|---|---|
| 1 | 0–3 h | NFAS (*n* = 30) | 9.87 ± 1.50[b] (100)[c] | 8.90 ± 1.40 (100) |
| | | FA18TO (*n* = 30) | 10.07 ± 1.76 (102) | 9.00 ± 1.49 (101) |
| | | XP6KO(A) (*n* = 30) | 2.80 ± 1.13 (28.4) | 1.43 ± 0.90 (16.1) |
| 2 | 0–3 h | NFAS (*n* = 50) | 6.50 ± 1.47 (100) | 6.92 ± 1.32 (100) |
| | | FA14TO (*n* = 50) | 6.06 ± 1.48 (93.2) | 7.22 ± 1.30 (104) |
| | | FA18TO (*n* = 50) | 5.68 ± 1.56 (87.4) | 7.38 ± 1.41 (113) |
| | | XP35KO(A) (*n* = 50) | 0.62 ± 0.78 (9.5) | 0.66 ± 0.72 (9.5) |
| 3 | 5–8 h | NFAS (*n* = 50) | 4.80 ± 1.64 (100) | 4.08 ± 1.45 (100) |
| | | FA18TO (*n* = 50) | 4.72 ± 1.44 (98.3) | 4.58 ± 1.49 (112) |
| | | XP35KO(A) (*n* = 50) | 0.58 ± 0.56 (12.1) | 0.52 ± 0.61 (15.0) |

[a]Exp. No. 1: 1 h-treatment with 0.22 mM DWA2114R or 0.0167 mM CDDP, followed by the initial 3 h labelling with [$^3$H]dThd (370 kBq/ml) in 2 mM HU and 14-day exposure. Exp. No. 2: 1 h-treatment with 2.2 mM DWA2114R or 0.167 mM CDDP, followed by the initial 3 h-labelling with [$^3$H]dThd (185 kBq/ml) in 2 mM HU and 9-day exposure. Exp. No. 3: 1 h-treatment with 0.44 mM DWA2114R or 0.05 mM CDDP, followed by a 5 h post-incubation with 2 mM HU, 3 h-labelling with [$^3$H]dThd (185 kBq/ml) in 2 mM HU and 12-day exposure. [b]Standard deviation of the mean. [c]The numerals in the parentheses indicate % UDS, relative to NFAS normal.

**Table II** Mean lethal doses (*Do*) of DWA2114R, carboplatin and CDDP

| Component of survival curve | Cell strain | *Do* ± *SD* (μM)[a] *DWA2114R* | *Carboplatin* | *CDDP* |
|---|---|---|---|---|
| First component | *Normal* | | | |
| | NFAS | 87.4 ± 3.98 | 79.0 ± 4.55 | 10.0 ± 0.00 |
| | TIG-1 | 88.0 ± 11.30 | 86.7 ± 5.70 | 9.93 ± 0.23 |
| | Mean[b] | 87.6 ± 5.65 | 82.3 ± 6.18 | 9.97 ± 0.15 |
| | *FA* | | | |
| | FA18TO | 25.4 ± 0.93 | 25.5 ± 0.71 | 3.90 ± 0.14 |
| | FA14TO | 25.3 ± 2.08 | 25.0 ± 1.00 | 3.13 ± 0.15 |
| | Mean | 25.4 ± 0.96 (0.29)[c] | 25.2 ± 0.83 (0.30) | 3.44 ± 0.44 (0.35) |
| | *XPA* | | | |
| | XP35KO | 48.8 ± 1.50 | 41.7 ± 2.89 | 3.93 ± 0.11 |
| | XP6KO | 45.8 ± 2.20 | 42.0 ± 3.61 | 3.70 ± 0.14 |
| | Mean | 47.1 ± 2.55[d] (0.54) | 41.8 ± 2.97[d] (0.51) | 3.84 ± 0.17 (0.39) |
| Second component | *Normal* | | | |
| | NFAS | 101.8 ± 5.37 | 96.3 ± 8.22 | 13.6 ± 1.40 |
| | TIG-1 | 102.5 ± 3.57 | 97.3 ± 7.02 | 13.4 ± 0.15 |
| | Mean | 102.0 ± 4.42 | 96.7 ± 7.11 | 13.5 ± 0.90 |
| | *FA* | | | |
| | FA18TO | 63.0 ± 2.08 | 75.0 ± 7.07 | 7.95 ± 0.07 |
| | FA14TO | 66.3 ± 1.50 | 72.3 ± 3.30 | 7.83 ± 0.45 |
| | Mean | 64.3 ± 2.73[e] (0.63) | 73.4 ± 4.78[e] (0.75) | 7.88 ± 0.33 (0.58) |
| | *XPA* | | | |
| | XP35KO | 80.7 ± 2.08 | 84.0 ± 5.29 | 8.87 ± 0.42 |
| | XP6KO | 80.3 ± 1.53 | 81.3 ± 8.05 | 9.50 ± 0.71 |
| | Mean | 80.5 ± 1.64 (0.79) | 81.5 ± 7.04 (0.84) | 9.12 ± 0.58 (0.68) |

[a]Mean *Do* ± SD of each cell strain from three to five times repeated survival curves. [b]Mean *Do* ± SD of the two strains each of normal, FA and XPA. [c]The numbers in parentheses are the FA/normal and XPA/normal ratios of *Do* of each agent. [d]$P = 0.005$ by Student's *t*-test. [e]$P = 0.003$ by Student's *t*-test.

crosslinks in ~70% DNA immediately after the 1 h treatment, followed by ~30% additional interstand crosslinking during the subsequent 5 h. With the doses of both agents, the 5 h post-incubation yielded the maximum of 8.5–10 interstrand crosslinks per $10^9$ Da in the FA18TO DNA *in vivo*, or ~1 crosslink unit per an ssDNA molecule in average size of $Mn_{(o)} = 5.57 \times 10^7$ [$= (\sim 1.1 \times 10^8/5.57 \times 10^7)-1$].

Figure 3 plots the concentration-dependent Pt(II) interstrand crosslinking in the FA18TO DNA. Rates of interstrand crosslinking immediately after the 1 h treatment were 1.7 crosslinks/$10^9$ Da per 1 mM DWA2114R (Figure 3a) and 4.7 crosslinks/$10^9$ Da per 0.1 mM CDPP (Figure 3b). Thus, DWA2114R is ~28 times less efficient than CDDP immediately after the 1 h treatment. Rates of the maximum interstrand crosslinking at 5 h of postincubation were 5.7 crosslinks/$10^9$ Da per 1 mM DWA2114R (Figure 3a), and 5.4 crosslinks/$10^9$ Da per 0.05 mM CDDP (Figure 3b). Thus, such a maximum rate with DWA2114R is also slower, and a concentration of DWA2114R 18 times that of CDDP, will be required for the production of equal numbers of interstrand crosslinks *in vivo* on the 1 mM basis.

### *Repair of interstrand crosslinks*

Figure 4 illustrates the time kinetics of early progressive formation of interstrand crosslinks and the subsequent repair in NFAS (normal), FA18TO and XP35KO(A) cells. As indicated above, the initial levels of interstrand crosslinks immediately after a 1 h treatment were low (~1.5/$10^9$ Da) for 2.2 mM DWA2114R (Figure 4a) and high (~6/$10^9$ Da) for 0.167 mM CDDP (Figure 4b) in all types of cells. Interstrand crosslinking progressed during the subsequent post-

**Figure 3** DWA2114R or CDDP concentration- and time-dependent interstrand crosslinking in FA18TO DNA. [$^3$H]dThd-labelled FA18TO cells were treated with the indicated concentrations of DWA2114R or CDDP in PBS for 1 h at 37°C, washed and further incubated in FBS-MEM for 5 h at 37°C to allow maximum crosslinking in cells. DNA was centrifuged as in Figure 2, and the number of interstrand crosslinks was plotted against concentration. Saturation of interstrand crosslinking occurred at ~1 crosslink unit per a $5.57 \times 10^7$ Da-ssDNA molecule [$= (\sim 1 \times 10^8)/(5.57 \times 10^7) - 1$]. ○——○, immediately after 1 h treatment; ●——●, 5 h post-incubation. **a**, DWA2114R: interstrand crosslinking rates immediately and 5 h after treatment were 1.7 and 5.7 crosslinks/$10^9$ Da/l mM, respectively; **b**, CDDP: interstrand crosslinking rates immediately and 5 h after treatment were 4.7/$10^9$ Da/0.1 mM and 5.4//$10^9$ Da/0.05 mM, respectively.

**Figure 4** Time dependent increase and one-arm unhooking of interstrand crosslinks. [$^3$H]dThd-labelled NFAS, FA18TO and XP35KO cells were treated with either 2.2 mM DWA2114R or 0.167 mM CDDP in PBS for 1 h, and incubated in MEM-FBS up to 24 h. The numbers of interstrand crosslinks remaining at the indicated post-incubation times were assessed by alkaline sucrose sedimentation (see Figure 2). **a**, DWA2114R; **b**, CDDP. O, NFAS normal; ●, FA18TO; ▲, XP35KO(A). The $t_{\frac{1}{2}}$ values for NFAS and XP35KO(A) cells in the 5 to 24 h period were 5 h **a** and 7 h **b**.

incubation for 5 h to give 5 and 1.5-fold different elevations for DWA2114R and CDDP, respectively, until reaching the similar maxima of 7–10 crosslinks/$10^9$ Da at 5 h in NFAS, FA18TO and XP35KO(A) cells (Figures 4a and b). Afterwards, both NFAS and XP35KO(A) cells performed the first half-excision of both DWA2114R and CDDP interstrand crosslinks at a similar rate of $t_{\frac{1}{2}}$ = 5–7 h (Figures 4a and b). On the contrary, FA18TO cells failed to unhook one arm of DWA2114R and CDDP interstrand crosslinks between 8 and 24 h of the post-incubation period, except for a 20–30% minor loss between 5 and 10 h by perhaps replicative dilution (Figures 4a and b). Thus, XPA cells were normal in the first step of interstrand crosslink repair, while FA cells failed in unhooking one arm of Pt(II) interstrand links.

### *Repair of intrastrand crosslinks by excision repair*

For UDS assay, cells were treated with DWA2114R (0.22, 0.44 and 2.2 mM) or CDDP (0.0167, 0.05 and 0.167 mM) for 1 h and then radioactivity labelled for 3 h with [$^3$H]dThd during the 0–3 h and 5–8 h intervals after treatment (see Table I, legend). The average numbers of Pt(II)-induced grains per nucleus in both intervals after DWA2114R and CDDP were small even in normal cells (Table I), as reconciled with poor repair of intrastrand crosslinks (see Introduction). The XPA (XP6KO, XP35KO) cells showed a lower level of 9.5–28% UDS of normal during both labelling intervals (Table I), indicating a defect of the XPA cells in excision repair of intrastrand crosslinks and monoadducts. However, FA14TO and FA18TO cells were normal in UDS (~100%) in both labelling intervals (Table I). Figure 4 and Table I indicate clearly that the FA cells have a defect in the repair of Pt(II) interstrand crosslinks, but the XPA cells have a defect in excision repair of Pt(II) intrastrand crosslinks.

### *Differential lethal effects of DWA2114R, carboplatin and CDDP*

Figures 5a to c show the biphasic clonogenic survival curves of 1 h-treated representative cells, for comparison of the lethal effects of DWA2114R, carboplatin and CDDP. Table II summarizes *Do* ± SD values of the two strains each of the normal, FA and XPA genotypes. All of the first components covered the major range of 80–90% kills (Figure 5). The following characteristics emerge from the first component *Do* values in Figure 5 and Table II. (i) The *Do* values of DWA2114R and carboplatin were similar and approximately an order of magnitude greater than those of CDDP in all types of cells (Figure 5 and Table II). (ii) The interstrand crosslink repair-defective FA cells and the intrastrand crosslink repair-defective XPA cells were hypersensitive to DWA2114R, carboplatin and CDDP (Figure 5), indicating that both inter- and intrastrand crosslinks are lethally acting. (iii) Toward CDDP (Figure 5c and Table II), the FA (mean *Do* = 3.44 ± 0.44 μM) and XPA cells (mean *Do* = 3.84 ± 0.17 μM) were similarly three times more sensitive than the normal (mean *Do* = 9.97 ± 0.15 μM). This balance of CDDP killing suggest, taking into account the alternative repair defects in the FA and XPA cells, that minor interstrand crosslinks are more lethal than abundant intrastrand crosslinks, when not repaired. (iv) Toward DWA2114R killing

**Figure 5** Clonogenic survival curves after a 1 h treatment with DWA2114R, carboplatin or CDDP. NFAS, FA18TO and XP35KO cells were treated with the indicated concentrations for 1 h at 37°C, washed, and incubated for 14 days for colony assay. **a**, DWA2114R; **b**, carboplatin; **c**, CDDP. O, NFAS normal (mean of 4 exps.; plating efficiencies (PE) = 18–26%); ●, FA18TO (mean of 3 exps.; PE = 10–15%); ▲, XP35KO(A) (mean of 2 exps.; PE = 19–25%). *Bar* indicates SD.

(Figure 5a and Table II), the FA cells (mean $Do = 25.4 \pm 0.96\,\mu M$) were most sensitive (3.5 times), and the XPA cells (mean $Do = 47.1 \pm 2.55\,\mu Mo$) were 1.9 times more sensitive than the normal cells (mean $Do = 87.6 \pm 5.65\,\mu M$). (v) Such situations were more or less similar for carboplatin (Figure 5b). Compared to the DWA2114R killing, however, the FA cells were slightly less sensitive to carboplatin ($P = 0.003$), while the XPA cells were slightly more sensitive to it ($P = 0.005$), including the second components (Figure 5 and Table II). Next, the second components of residual survival (<20%) for all types of cells were less steep than the first (Figure 5 and Table II). Since the number of interstrand crosslinks (presumably total Pt(II)-adducts to DNA) increases as a linear function of concentration (Figure 3), the less steep second components (Figure 5) suggest that the excess numbers of inter- and intrastrand crosslinks by $\geqslant 70\,\mu M$ DWA2114R and carboplatin or $\geqslant 7\,\mu M$ CDDP may be less lethally acting. Nonetheless, those lethal effects of the three agents followed a roughly similar tendency as described above for the first components.

Further, we studied the differential lethal effects of continuous treatment with a fixed low dose of $44\,\mu M$ DWA2114R or $3.33\,\mu M$ CDDP (approximately *Do* of XPA in Table II). Figure 6 shows a biphasically increasing lethal effects with time, indicating that DWA2114R and CDDP exerted 90% or more lethal effects within the initial 6 h. Again, FA18TO cells were most sensitive, and XP35KO(A) cells were intermediately more sensitive to DWA2114R than normal cells (Figure 6a). Such a treatment with CDDP resulted in the balanced killing of FA and XPA cells (Figure 6b). In conclusion, FA cells were more sensitive to DWA2114R and carboplatin than XPA cells.

**Figure 6** Differential lethal effects of continuous treatment with DWA2114R or CDDP. NFAS, FA18TO and XP35KO(A) cells were treated continuously up to 24 h with $44\,\mu M$ DWA2114R or $3.33\,\mu M$ CDDP in FBS-free MEM, washed and incubated in FBS-MEM for 14 days for colony assay. Each time point of treated cell survival was corrected for reduction in plating efficiencies (0–15%) during the 0 to 24 h incubation in FBS-free medium. **a**, DWA2114R; **b**, CDDP. ○, NFAS normal; ●, FA18TO; ▲, XP35KO(A).

### Discussion

First, the present results have indicated the differential interstrand crosslinking of genomic DNA of human cells by DWA2114R and CDDP. DWA2114R with CBDCA (Figure 1) was less monoadducting to DNA, judged by a low yield of the final interstrand crosslinks (Figure 3), as carboplatin with hydrolysis-resistant CBDCA (Figure 1) showed two orders magnitude slower rate of *in vitro* DNA platination that did CDDP (Knox *et al.*, 1986). The rate of CDDP interstrand crosslinking was faster as accounted for by a 60–70% yield immediately after 1 h treatment (Figures 2 to 4), as described previously (Roberts & Friedlos, 1987). Most of the early 1 h-products with DWA2114R were monoadducts (Figure 3), while once they were formed, interstrand crosslinking progressed more rapidly ($t_{½} \approx 2$ h for pre-crosslinks) in the normal, FA and XPA cells (Figure 4). Maximum interstrand crosslinking of DNA in cells with both agents was observed at 5 h of the post-incubation period (Figure 4), as found with CDDP (Eastman, 1985; Knox *et al.*, 1986; Fujiwara *et al.*, 1987). The maximum rate indicated that a molar concentration of DWA2114R 18 times that of CDDP was required for producing the equal numbers of interstrand crosslinks (Figure 3). Thus, the common hydrolysis-resistant CBDCA group in both carboplatin and DWA2114R (Figure 1) is rate-limiting in the DNA adduction. The *in vitro* binding rate of the second Pt(II) arm of carboplatin has been shown to be slow with $t_{½} = 13$ h (Knox *et al.*, 1986; Roberts & Friedlos, 1987). However, the interstrand crosslinking rate *in vivo* of carboplatin (Knox *et al.*, 1986) and DWA2114R ($t_{½} = 2$ h in Figure 4) approaches that of CDDP *in vivo*. Thus, the CBDCA leaving group becomes more labile after monoadducting to DNA.

We studied the repair of Pt(II) inter- and intrastrand crosslinks. Interstrand crosslink repair is a two-step process involving the first half excision of one arm of Pt(II) interstrand crosslinks and the subsequent removal of half-excised monoadducts (Fujiwara *et al.*, 1977; Fujiwara, 1983). Unilateral arms of DWA2114R or CDDP-induced Pt(II) interstrand crosslinks were unhooked with $t_{½} = 5–7$ h in normal cells (Figure 4). This $t_{½}$ is three times slower than that (= 2 h) for MC interstrand crosslinks (Fujiwara, 1982). This difference could arise from different accessibility of repair enzymes to Pt(II) and MC interstrand crosslinks. The FA cells (FA18TO, FA14TO) were also almost completely defective in the first half excision of Pt(II) interstrand crosslinks, but XPA cells were proficient in that process (Figure 4) (Fujiware *et al.*, 1987). Excision repair of Pt(II) instrastrand crosslinks in mammalian cells is poor (Ciccarelli *et al.*, 1985; Heiger-Bernays *et al.*, 1990). Particularly, the major d(GpG) intrastrand crosslinks are refractory to excision repair by normal human cell extract (Szymkowski *et al.*, 1992). Thus, a small amount of repair synthesis may arise by other intrastrand lesions. Furthermore, the amount of UDS induced by abundant intrastrand crosslinks in proficient normal and FA cells was limited (Table I), as previously indicated with MC (Fujiwara *et al.*, 1977) and CDDP (Plooy *et al.*, 1985). XPA had a 5–10-fold reduced UDS after DWA2114R and CDDP (Table I), as observed in repair synthesis in platinated M13 DNA using the XPA cell extract (Szymkowski *et al.*, 1992).

The lethal effect was different between DWA2114R or carboplatin and CDDP. The equi-toxicity in terms of *Do* required a concentration of DWA2114R or carboplatin an order of magnitude greater due to reductions in DNA adducting (see above) and pharmacodynamic uptake into cells (Akamatsu *et al.*, 1991; Knox *et al.*, 1986), than that of CDDP (Figure 5 and Table II). Further, in phase with the maximum crosslinking at 5 h (Figure 4), more than 90% kills of FA and XPA cells by DWA2114R and CDDP were attained within 6 h (Figure 6), indicating the tight association with early production of 90% or more lethally acting DNA lesions. The killing efficiencies of Pt(II) inter- and intrastrand crosslinks are also different. *Do* of CDDP was similar between intrastrand crosslink repair-defective XPA and interstrand crosslink repair-defective FA cells (Table II), indicating that the abundant former and the minor latter lesions remaining unrepaired exert the balanced killing, which is reflective of a higher killing efficiency of interstrand crosslink. In addition, preferential repair in actively transcribed genes is not so distinct for both Pt(II) inter- and intrastrand crosslinks (Jones *et al.*, 1991). The completely excision-defective XPA cells were not so sensitive (at most 2-fold) to DWA2114R, carboplatin and CDDP (Figure 5) as to UV (Fujiwara *et al.*, 1977). Similarly, the XPA cells exhibited only a 1.5 to 2 times slight hypersensitivity to MC and monofunctional decarbamoyl mitomycin C, despite the 30-

fold MS supersensitivity of the FA cells by effective blocks to replication by interstrand crosslinks (Fujiwara *et al.*, 1977; Kano & Fujiwara, 1981). Amounts of MC and Pt(II) interstrand crosslinks in the cell DNA are 2–10%, compared to 90% or more intrastrand crosslinks. Therefore, the XPA cells can tolerate a great amount of monoadducts and intrastrand crosslinks by the replication-dependent or postreplication repair pathway, even though the major d(GpG) intrastrand crosslinks effectively block replication *in vitro* (Ciccarelli *et al.*, 1985; Heiger-Bernays *et al.*, 1990; Lemaire *et al.*, 1991; Iwata *et al.*, 1991). In consequence, interstrand crosslinks are more potentially lethal than intrastrand adducts.

In the first component of survival curves, low doses of DWA2114R killed more FA cells than XPA cells (Figures 5 and 6). This particular aspect can be discussed further, taking into account that the hypersensitive lethal fractions in FA and XPA cells arise by unrepaired inter- and intrastrand crosslinks, respectively. First, the relative lethal effects in terms of the FA/normal ratios of the first component *Do* are identically 0.3 for DWA2114R, carboplatin and CDDP (Table II). Thus, the different *Do* concentrations of the three agents will produce a single hit, or the identical lethal number of interstrand crosslinks in the DNA of FA cells. However, the XPA/normal ratios are different: 0.54 for DWA2114R, 0.51 for carboplatin and 0.39 for CDDP (Table II). These figures suggest that the number of intrastrand crosslinks per lethal hit in the XPA cells is less for DWA2114R and carboplatin than CDDP. Alternatively, it is suggested that, on the basis of a single lethal hit (*Do*), a relative ratio of highly lethally acting interstrand crosslinks over intrastrand crosslinks is greater with DWA2114R and carboplatin than CDDP.

Finally, the structural modification with CBDCA in DWA2114R and carboplatin may play a similar role in the reduction of mainly the pharmacodynamic uptake and DNA binding, but the 2-aminomethylpyrrolidine moiety in DWA2114R may not greatly affect DNA-adducting. Instead, the 2-aminomethylpyrrolidine moiety in DWA2114R would be related to a greater improvement of nephrotoxicity and myelosuppression, compared to carboplatin without that particular carrier ligand.

This work was supported by a Grant-in-Aid for Cancer Research from the Ministry of Education, Science and Culture, Japan. We thank Chugai Pharmaceutical Co. for generous gift of DWA2114R, and Drs M.S. Sasaki and T. Kaji for the FA and TIG-1 cells.

### References

AKAMATSU, K., ENDOH, K., MATSUMOTO, T., MORIKAWA, K., KOIZUMI, M., KOIZUMI, K. & MITSUI, H. (1991). Toxicological and tumoricidal evaluations of a new platinum complex, (−)-(R)-2-aminomethylpyrrolidine(1,1-cyclobutanedicarboxylato) platinum(II) monohydrate, in rats. *Jpn. J. Cancer Res.*, **82**, 724–731.

BEDFORD, P., FICHTINGER-SHEPMAN, A.M.J., SHELLARD, S.A., WALKER, M.C., MASTERS, J.R.W. & HILL, B.T. (1988). Differential repair of Pt-DNA adducts in human bladder and testicular tumor continuous cell lines. *Cancer Res.*, **48**, 3019–3024.

CICCARELLI, R.B., SOLOMON, M.J., VARSHAVSKY, A. & LIPPARD, S.J. (1985). *In vivo* effects of *cis*- and *trans*-diaminedichloroplatinum(II) on SV40 chromosomes: differential repair, DNA-protein cross-linking, and inhibition of replication. *Biochemistry*, **24**, 7533–7540.

CORDA, Y., JOB, C., ANIN, M.-F., LENG, M. & JOB, D. (1991). Transcription by eukaryotic and prokaryotic RNA polymerases of DNA modified at a d(GG) or a d(AG) site by antitumor drug *cis*-diaminedichloroplatinum(II). *Biochemistry*, **30**, 222–230.

EASTMAN, A. (1985). Interstrand cross-links and sequence specificity in the reaction of *cis*-dichloro(ethylenediamine)platinum(II) with DNA. *Biochemistry*, **24**, 5027–5032.

EASTMAN, A. (1987). The formation, isolation and characterization of DNA adducts produced by anticancer platinum complexes. *Pharmacol. Ther.*, **34**, 155–166.

ENDOH, K., AKAMATSU, K., MATSUMOTO, T., MORIKAWA, K., HONDA, M., MITSUI, H., KOIZUMI, M., KOIZUMI, K. & MATSUNO, T. (1989). Antitumor activity of a new platinum complex, 2-aminomethylpyrrolidine(1,1-cyclobutanedicarboxylato)platinum (II). *Anticancer Res.*, **9**, 987–992.

FICHTINGER-SCHEPMAN, A.M.J., VAN DER VEER, J.L., DEN HARTLOG, J.H.J., LOHMAN, P.H.M. & REEDIJK, J. (1985). Adducts of the antitumor drug *cis*-diaminodichloroplatinum(II) with DNA: formation, identification and quantitation. *Biochemistry*, **24**, 707–713.

FOSTER, B.J., HARDING, B.J., WOLPERT-DEFILLPPES, M.K., RUBINSTEIN, L.Y., CLAGETT-CARR, K. & LEYLAND-JONES, B. (1990. A strategy for the development of two clinically active cisplatin analogs: CBDCA and CHIP. *Cancer Chemother. Pharmacol.*, **25**, 395–404.

FUJIWARA, Y. (1982). Defective repair of mitomycin C cross-links in Fanconi's anemia and loss in confluent normal and xeroderma pigmentosum cells. *Biochim. Biophys. Acta*, **699**, 217–225.

FUJIWARA, Y. (1983). Measurement of interstrand cross-links produced by mitomycin C. In *DNA Repair – A Laboratory Manual of Research Procedures*, Friedberg, E.C. & Hanawalt, P.C. (eds), Vol. 2, pp. 143–160. Marcel Dekker, Inc., New York.

FUJIWARA, Y. (1989). Clustered repair of excisable 4-nitroquinoline-1-oxide adducts in a larger fraction of genomic DNA of xeroderma pigmentosum complementation group C cells. *Carcinogenesis*, **10**, 1777–1785.

FUJIWARA, Y., MATSUMOTO, A., ICHIHASHI, M. & SATOH, Y. (1987). Heritable disorders of DNA repair: xeroderma pigmentosum and Fanconi's anemia. *Curr. Probl. Dermatol.*, **17**, 182–198.

FUJIWARA, Y., TATSUMI, M. & SASAKI, M.S. (1977). Cross-link repair in human cells and its possible defect in Fanconi's anemia cells. *J. Mol. Biol.*, **113**, 635–649.

GILDSTEIN, R.S. & MAYOR, G.H. (1985). The nephrotoxicity of cisplatin. *Life Sci.*, **32**, 685–690.

HARRAP, K.R., JONES, M., WILKINSON, C.R., CLINK, H.M., SPARROW, S., MITCHLEY, B.C.V., CLARKE, S. & VEASEY, A. (1980). Antitumor, toxic and biochemical properties of cisplatin and eight other platinum complexes. In *Cisplatin: Current Status and New Developments*, Prestyko, A.W., Crooke, S.T. & Carter, S.K. (eds), pp. 193–212. Academic Press, Inc.: New York.

HEIGER-BERNAYS, W.J., ESSIGMANN, J.M. & LIPPARD, S.L. (1990). Effect of the antitumor drug *cis*-diaminedichloroplatinum(II) and related platinum complexes on eukaryotic DNA replication. *Biochemistry*, **29**, 8461–8466.

IWATA, M., IZUTA, S., SUZUKI, M., KOJIMA, K., FURUHASHI, Y., TOMODA, Y. & YOSHIDA, S. (1991). Sequence-dependent termination of mammalian DNA polymerase reaction by a new platinum compound, (−)-(R)-2-aminomethylpyrrolidine(1,1-cyclobutanedicarboxylato)platinum(II) monohydrate. *Jpn. J. Cancer Res.*, **82**, 433–439.

JONES, J., ZHEN, W., REED, E., PARKER, P.J., SANCAR, A. & BOHR, V.A. (1991). Gene-specific formation and repair of cisplatin intrastrand adducts and interstrand cross-links in Chinese hamster ovary cells. *J. Biol. Chem.*, **266**, 7101–7107.

KANO, Y. & FUJIWARA, Y. (1981). Roles of DNA interstrand cross-linking and its repair in the induction of sister-chromatid exchange and a higher induction in Fanconi's anemia cells. *Mutation Res.*, **81**, 365–375.

KNOX, R.J., FRIEDLOS, F., LYDALL, D.A. & ROBERTS, J.J. (1986). Mechanism of cytotoxicity of anti-cancer platinum drugs: evidence that *cis*-diaminedichloroplatinum(II) and *cis*-diamine (1,1-cyclobutanedicarboxylato)platinum(II) differ only in the kinetics of their interaction with DNA. *Cancer Res.*, **46**, 1972–1979.

LEMAIRE, M.-A., SCHWARTZ, A., RAHMOUNI, A.R. & LENG, M. (1991). Interstrand cross-links are preferentially formed at the d(GC) sites in the reaction between *cis*-diaminedichloroplatinum (II) and DNA. *Proc. Natl Acad. Sci. USA*, **88**, 1982–1985.

LEPRE, C.A. & LIPPARD, S.J. (1990). Interaction of platinum antitumor compounds with DNA. *Nucl. Acids Mol. Biol.*, **4**, 9–38.

MATSUMOTO, T., ENDOH, K., AKAMATSU, K., KAMISANGO, K., MITSUI, H., KOIZUMI, K., MORIKAWA, K., KOIZUMI, M. & MATSUMOTO, T. (1991). Comparison of the antitumor effects and nephrotoxicity-inducing activities of two new platinum complexes, (−)-(R)-2-aminomethylpyrrolidine(1.1-cyclobutanedicarboxylato)platinum(II) monohydrate and its enantiomeric isomer. *Br. J. Cancer*, **64**, 41–46.

MORIKAWA, K., HONDA, M., ENDOH, K., MATSUMOTO, T., AKAMATSU, K., MITSUI, M. & KOIZUMI, M. (1990). Synthesis of platinum complexes of 2-aminomethylpyrrolidine derivatives for use as carrier ligands and their antitumor activities. *Chem. Pharm. Bull.*, **38**, 930–935.

PAGE, J.D., HUSAIN, I., SANCAR, A. & CHANEY, S.G. (1990). Effect of the diaminocyclohexane carrier ligand on platinum adduct formation, repair and lethality. *Biochemistry*, **29**, 1016–1024.

PLOOY, A.C.M., VAN DIJK, M., BERENDS, F. & LOHMAN, P.H.M. (1985). Formation and repair of DNA interstrand cross-links in relation to cytotoxicity and unscheduled DNA synthesis induced in control and mutant human cells treated with *cis*-diaminedichloroplatinum(II). *Cancer Res.*, **45**, 4178–4184.

ROBERTS, J.J. & FRIEDLOS, F. (1987). Quantitative estimation of cisplatin-induced DNA interstrand cross-links and their repair in mammalian cells: relationship to toxicity. *Pharmacol. Ther.*, **34**, 215–246.

ROSENBERG, B., VAN CAMP, L., TROSKO, J.E. & MANSOUR, V.H. (1969). Platinum complexes: a new class of potent antitumor agents. *Nature*, **222**, 385–386.

SORENSON, C.M. & EASTMAN, A. (1988). Mechanism of *cis*-diaminedichloroplatinum(II)-induced cytotoxicity:. role of G2 arrest and DNA double-strand breaks. *Cancer Res.*, **48**, 4484–4488.

SZYMKOWSKY, D.LE., YAREMA, K., ESSIGMANN, J.M., LIPPARD, S.J. & WOOD, R.D. (1992). An intrastrand d(GpG) platinum crosslink in duplex M13 DNA is refractory to repair by human cell extracts. *Proc. Natl Acad. Sci. USA*, **89**, 19772–10776.

VON HOFF, D.D., SCHILSKY, R., REICHART, C.M., REDDICK, R.L., ROZENCWIG, M., YOUNG, R.C. & MUGIA, F.M. (1979). Toxic side effects of *cis*-dichlorodiamineplatinum(II) in man. *Cancer Treat, Rep.*, **63**, 1527–1531.